# Sputum Bacterial Metacommunities in Distinguishing Heterogeneity in Respiratory Health and Disease

**DOI:** 10.3389/fmicb.2022.719541

**Published:** 2022-03-31

**Authors:** Jiyeon Si, Yongbin Choi, Jeroen Raes, Gwangpyo Ko, Hyun Ju You

**Affiliations:** ^1^Medical Science Research Institute, School of Medicine, Sungkyunkwan University (SKKU), Suwon, South Korea; ^2^Department of Environmental Health Sciences, Graduate School of Public Health, Seoul National University, Seoul, South Korea; ^3^Center for Human and Environmental Microbiome, Seoul National University, Seoul, South Korea; ^4^Department of Microbiology and Immunology, Rega Institute for Medical Research, Leuven, Belgium; ^5^VIB-KU Leuven Center for Microbiology, Leuven, Belgium; ^6^Bio-MAX/N-Bio, Seoul National University, Seoul, South Korea; ^7^KobioLabs, Seoul, South Korea; ^8^Institute of Health and Environment, Seoul National University, Seoul, South Korea

**Keywords:** sputum microbiome, community typing, *Prevotella*, metacommunity, inflammation, network, COPD metacommunity in sputum microbiome

## Abstract

**Background and Objective:**

Cluster-based analysis, or community typing, has been attempted as a method for studying the human microbiome in various body niches with the aim of reducing variations in the bacterial composition and linking the defined communities to host health and disease. In this study, we have presented the bacterial subcommunities in the healthy and the diseased population cohorts and have assessed whether these subcommunities can distinguish different host health conditions.

**Methods:**

We performed community typing analysis on the sputum microbiome dataset obtained from a healthy Korean twin-family cohort (*n* = 202) and an external chronic obstructive pulmonary disease (COPD) cohort (*n* = 324) and implemented a networks analysis to investigate the associations of bacterial metacommunities with host health parameters and microbial interactions in disease.

**Results:**

The analysis of the sputum microbiome of a healthy Korean cohort revealed high levels of interindividual variation, which was driven by two dominant bacteria: *Neisseria* and *Prevotella*. Community typing of the cohort samples identified three metacommunities, namely, *Neisseria* 1 (N1), *Neisseria* 2 (N2), and *Prevotella* (P), each of which showed different functional potential and links to host traits (e.g., triglyceride levels, waist circumference, and levels of high-sensitivity C-reactive protein). In particular, the *Prevotella*-dominant metacommunity showed a low-community diversity, which implies an adverse health association. Network analysis of the healthy twin cohort illustrated co-occurrence of *Prevotella* with pathogenic anaerobic bacteria; this bacterial cluster was negatively associated with high-density lipoproteins but positively correlated with waist circumference, blood pressure, and pack-years. Community typing of the external COPD cohort identified three sub-metacommunities: one exclusively comprising healthy subjects (HSs) and the other two (CS1 and CS2) comprising patients. The two COPD metacommunities, CS1 and CS2, showed different abundances of specific pathogens, such as *Serratia* and *Moraxella*, as well as differing functional potential and community diversity. Network analysis of the COPD cohort showed enhanced bacterial coexclusions in the CS metacommunities when compared with HS metacommunity.

**Conclusion:**

Overall, our findings point to a potential association between pulmonary *Prevotella* and host health and disease, making it possible to implement community typing for the diagnosis of heterogenic respiratory disease.

## Introduction

Advances in high-throughput sequencing in the field of human microbiome research have expanded our knowledge of host–microbe interactions and their effects on human health and disease. The respiratory tract, which has one of the largest surface areas in the human body (70 m^2^) (Huffnagle et al., 2017), provides a distinct ecological habitat for microbes. In particular, the upper respiratory tract (nasal and oral passages), which connects the external environment to the deeper lower airways, undergoes constant interaction with inhaled microbes and resident bacteria. Furthermore, because sputum samples can be collected in a non-invasive way, researchers have shown interest in investigating the microbiome of the upper respiratory tract to assess its value as a biomarker for lung cancer, tuberculosis, asthma, cystic fibrosis, and chronic obstructive pulmonary disease (COPD) (Krishna et al., 2016; Cameron et al., 2017; Leitao Filho et al., 2019; Pang et al., 2019; Quinn et al., 2019). Of the pulmonary diseases, COPD has had the fourth highest global mortality rate since 2015 (Sidhaye et al., 2018). The unique characteristics of this disease stem from its clinical heterogeneity with respect to symptoms, progression, and survival; indeed, there are up to seven different phenotypes of the disease (Han et al., 2010; Mirza and Benzo, 2017). Because of this complexity, medical treatment is still at an early stage, making prevention or rapid diagnosis prior to disease exacerbation the best treatment strategy.

The human microbiome harbors high levels of interindividual variation, which complicates linkage with host features. To separate disease-relevant/-specific signals linked to the microbiome from the background noise caused by interindividual variations, attempts have been made to stratify host populations in the gut, vagina, lung, and saliva microbiomes according to community composition (Arumugam et al., 2011; Ravel et al., 2011; Segal et al., 2013; Xun et al., 2018). In particular, a unique microbial cluster in the gut has biological linkages with intestinal activity, host health, and disease (Vandeputte et al., 2017; Valles-Colomer et al., 2019; Vieira-Silva et al., 2019). Therefore, to find out whether the highly variable sputum microbiota can be grouped and connected to host health status, we first implemented community typing of 202 sputum microbiome sequencing data from the Healthy Twin Study (Sung et al., 2006) and identified three distinct metacommunities. Interestingly, a *Prevotella*-dominant metacommunity with the lowest community diversity showed potential associations with smoking, hsCRP, triglyceride (TG) levels, and waist circumference. Next, we hypothesized that patients with COPD would harbor multiple metacommunities, in line with the heterogenous disease traits of COPD. The same community typing approach applied to an independent COPD cohort identified two sub-metacommunities showing distinct functional differences. Taken together, this study provides evidence of a potential link between *Prevotella* and host (pulmonary) health and raise the possibility that community typing can help in distinguishing between heterogeneous host respiratory health disorders, making it useful for prognostic or diagnostic purposes.

## Materials and Methods

### Study Cohorts

Sputum sequencing data generated from the Healthy Twin Study in Korea were used for this study (European Nucleotide Archive; ERP010734) (Sung et al., 2006; Lim et al., 2016). Subjects were excluded from this study if they had received antibiotic treatment or cold medication within the past 3 months. Also, subjects with symptoms of airway diseases such as asthma or COPD were excluded. The sequencing data were further filtered to ensure that only subjects aged 30–60 years old were included. A gender ratio was balanced using the optmatch R package (Hansen and Klopfer, 2006). Consequently, sequencing data from 202 participants (including 53 monozygotic and 12 dizygotic twin pairs and their family members) and their associated metadata were analyzed. Additional sputum sequencing data from an external COPD cohort (*n* = 324) (Haldar et al., 2020) were obtained from the Sequence Read Archive [under accession numbers PRJNA491861 (healthy samples, *n* = 121) and SRP102480 (COPD samples, *n* = 203)].

### 16S rRNA Sequence Data Analysis

Raw sequence data were processed further using QIIME (version 1.8.0) (Kuczynski et al., 2011). Operational taxonomic unit (OTU) picking was performed by clustering sequences at 97% similarity. Taxonomic classification of each OTU was performed using the Ribosomal Database Project classifier (Wang et al., 2007) and the Greengenes database (version 13_5) (DeSantis et al., 2006). An OTU count table was then agglomerated at the genus level before downstream analysis. Sequence files from the COPD cohort were analyzed following DADA2 pipelines (1.12.1) using default settings (Callahan et al., 2016). The trim parameters for the healthy samples were as follows: sequence truncate 200 bp for both the forward and reverse sequence reads; trim left 10. Trim parameters for patients with COPD were as follows: sequence truncate 150 bp for forward and 130 bp for reverse sequence reads; trim left 10.

### Functional Potential of the Sputum Microbiota

The functional potential of the sputum microbiota was predicted using Piphillin based on the 16s rRNA sequences (Iwai et al., 2016). Piphillin infers metagenomic functions using OTU counts and their representative sequences; it then matches the sequences to the nearest neighbor in the genome database. Prior to analysis, OTUs observed in less than 50% of samples were excluded. A 95% identity cut-off was used to determine the similarity between 16s rRNAs and the reference genome database. Amplicon sequence variant sequences obtained for the COPD cohort were implemented at a 97% identity cutoff. For functional analysis, the Kyoto Encyclopedia of Genes and Genomes (KEGG) database was used at the pathway level.

### Identification of Bacterial Clusters

The weighted correlation network analysis (WGCNA) was performed to determine bacterial interactions with the host (Langfelder and Horvath, 2008). This software implements a signed weighted co-occurrence network in which the negatively connected nodes are considered unconnected. Genera observed in < 50% of samples were excluded to reduce false positives due to low abundance taxa; data were then subjected to Hellinger transformation and were log-scaled. Soft-thresholding power (β = 3), which satisfies the scale-free topology of the network, was determined using the *pickSoftThreshold* function. A signed adjacency matrix was then converted to a Topological Overlap Matrix and used to define bacterial clusters based on hierarchical clustering (average linkage). Association of the bacterial clusters with clinical variables was determined by correlating the eigenvector of each cluster with host variables.

### Network Analysis to Determine Microbe–Microbe Interactions

To investigate microbe–microbe interactions, network analysis was performed using SparCC, with 500 bootstraps used to estimate *p* values (Friedman and Alm, 2012). A *Q*-value < 0.002 was considered significant. Taxa comprising < 20% of the population were excluded. Final networks were plotted using Cytoscape (version 3.7.2; perfused force directed layout) (Shannon et al., 2003).

### Statistical Analysis

After randomly rarefied to 9,000 reads, microbial diversity was determined using the *phyloseq* and *vegan* R packages (Dixon, 2003; McMurdie and Holmes, 2013). The COPD cohort samples were rarefied to 10,000 reads. Community variation was determined by nonmetric non-metric multidimensional scaling and principal coordinates analysis using the *metaMDS* and *envfit* functions in the *vegan* package. Identification of metacommunities was performed by fitting the models of Dirichlet multinomial mixtures to the community structure (Holmes et al., 2012). The number of metacommunities was determined as the number that provides the minimum Laplace approximation to the model evidence. Associations between metacommunities and clinical variables were analyzed using the Wilcoxon rank sum test, the Kruskal–Wallis test followed by the Dunn’s *post hoc* test (for more than the two groups), or the chi-square test with *post hoc* tests. A pairwise Adonis test was performed using the *pairwiseAdonis* package (Martinez Arbizu, 2020). When testing more than two parameters, all the statistical tests were followed by multiple testing correction using the Benjamini–Hochberg method (denoted as a *q* value). All the analyses were conducted using R (version 3.4.1).

## Results

### Metacommunity of the Sputum Microbiota in a Healthy Korean Twin-Family Cohort

**FIGURE 1:**
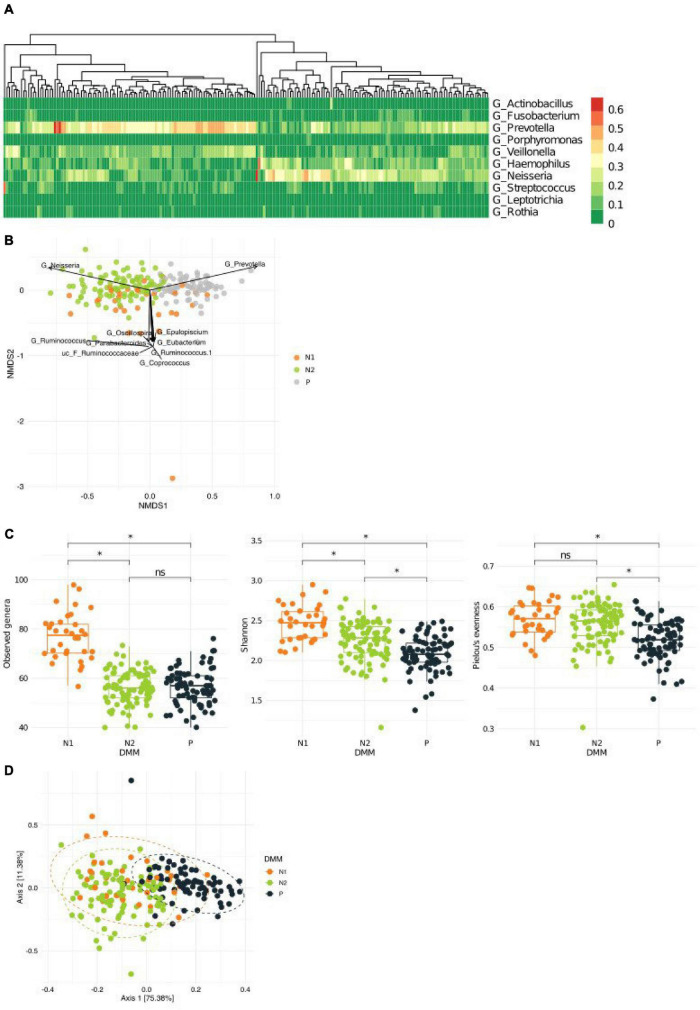
Variations in the microbiota in sputum samples collected from a healthy Korean cohort. **(A)** Heatmap showing the top 10 most abundant bacteria in the sputum microbiota. **(B)** A non-metric multidimensional scaling (NMDS) plot in which metacommunities are assigned a color. Arrows indicate the top 10 bacteria contributing to community variation. **(C)** Metacommunity diversity at the genus level. **(D)** Different inferred functional characteristics of the sputum microbiome among metacommunities. A principal coordinates analysis (PCoA) plot showing the distribution of functional features of metacommunity. *q < 0.001.

Despite the high degree of interindividual variation, we found distinct patterns of dominant genera in the sputum microbiota of the healthy Korean twin-family cohort (*n* = 202; Figure 1A). To analyze whether these patterns allow stratification of the cohort, we clustered the airway microbiota by fitting the taxonomic abundances to a probabilistic model fitting a Dirichlet multinomial mixture, which gives the optimal number of clusters by modeling sparse microbiome data (Supplementary Figure 1A). This process identified three metacommunities, in which the dominant bacteria were *Prevotella* and *Neisseria* (Supplementary Figures 1B, C). Because these two genera were the major driving taxa for two of the three clusters (the other cluster was driven by multiple taxa) on an NMDA plot, we defined clusters according to predominance; however, we subclassified the *Neisseria*-dominant clusters into N1 and N2 based on *Neisseria* abundance (Figure 1B). Microbial composition at the phylum level revealed more abundant Proteobacteria in the N1 and N2 metacommunities than in the *Prevotella*-dominant metacommunity, while Bacteroidetes and Firmicutes showed the opposite pattern (Supplementary Figure 1D). Community diversity analysis showed that the *Prevotella* (P) metacommunity had significantly lower richness and evenness than the other subtypes (the Kruskal–Wallis test, *q* < 0.1; Figure 1C). Because the metacommunities in the sputum microbiota showed compositional differences, we further analyzed them to evaluate whether they also showed functional differences. Functional potential was inferred based on 16S rRNA sequence data, which showed distinct functional clustering of the *P* metacommunity (Figure 1D and Supplementary Table 1). More than 35% of the community variation could be explained by the *P* metacommunity associating with the other *N* subtypes. However, the *N* metacommunities together explained only 4% of community variation, suggesting homogeneity Between *N* metacommunities with regard to their functions.

### Link Between Metacommunities, Host Health, and Smoking Status

**FIGURE 2:**
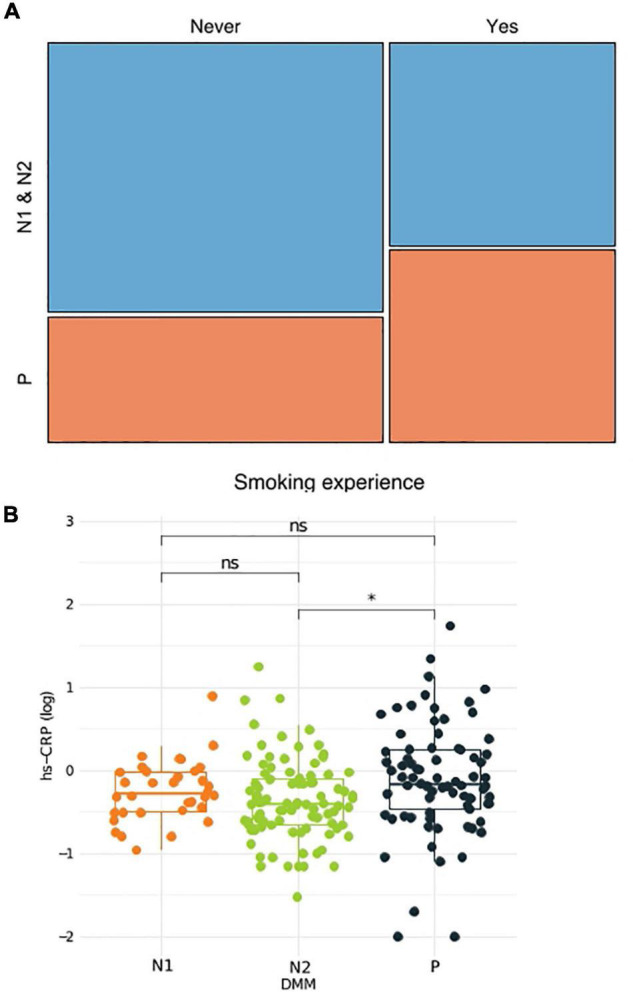
Association between the host health status and metacommunity. **(A)** Subdivision of a metacommunity and the association between each subcommunity and host smoking experience. **(B)** A box plot and a whisker plot showing the 25th percentile, the median, the 75th percentile, and minimum and maximum data points. *q < 0.01.

To better understand the link between metacommunities and host health status, we analyzed the association between metabolic parameters and the metacommunities (Table 1). We found that TG levels and waist circumference were significantly different among individuals with different metacommunities: the *P* metacommunity was associated with significantly greater TG levels and waist circumference than the N2 community (the Kruskal–Wallis test, *q* < 0.1; Supplementary Figure 2A). Further taxonomic association analyses revealed that, out of the top six most prevalent genera in the metacommunities, *Haemophilus* and *Prevotella* showed the most significant associations with clinical characteristics (Supplementary Figure 2B). In addition, we analyzed the association between the host smoking status and different metacommunities. The number of current smokers was not significantly different among metacommunities (chi-squared test, *p* > 0.05; Supplementary Figure 3A). However, we observed an increasing trend between smoking and the *P* metacommunity after clustering of the *N* metacommunities (chi-squared test, *p* = 0.052; Supplementary Figure 3B). Further clustering of smokers by “ever-smoking experience” led to even more marked distinction between the *P* metacommunity and the other two (chi-squared test, *p* = 0.032; Figure 2A and Supplementary Table 2). The significant increase in hsCRP levels in those with the *P* metacommunity indicated a potential association between this metacommunity and the host inflammatory status (the Kruskal–Wallis test, *q* < 0.1; Figure 2B).

**TABLE 1:** Demographic and clinical metadata in a metacommunity.

		Metacommunity (mean ± s.d.)			
	Total	N1	N2	*P*	*q*-value
Sex					
Male	101	11	42	48	
Female	101	23	49	29	
Total	202	34	91	77	
Age	45.56 ± 10.19	46.68 ± 10.44	44.23 ± 8.82	46.64 ± 11.45	
BMI	23.68 ± 3.17	22.86 ± 2.15	23.38 ± 3.12	24.39 ± 3.47	
FBS (mg/dL)	100.10 ± 33.88	95.97 ± 16.97	95.20 ± 20.62	107.71 ± 48.09	
HDL (mg/dL)	49.89 ± 12.76	52.79 ± 13.11	50.68 ± 13.62	47.68 ± 11.26	
TG (mg/dL)	135.20 ± 92.51	119.15 ± 53.03	121.75 ± 88.25	158.19 ± 106.40	*
Waist (cm)	81.34 ± 9.51	78.76 ± 6.47	80.01 ± 9.07	84.04 ± 10.55	*
hsCRP (mg/L)	1.56 ± 4.56	0.87 ± 1.31	0.94 ± 2.12	2.58 ± 6.87	*
SBP (mm Hg)	114.31 ± 15.02	114.06 ± 16.85	112.41 ± 13.54	116.68 ± 15.68	
DBP(mm Hg)	71.54 ± 10.41	73.12 ± 8.79	70.00 ± 10.10	72.68 ± 11.27	

*s.d., standard deviation. FBS, fasting blood sugar; HDL, High-density lipoprotein cholesterol; TG, triglyceride; Waist, waist circumference; hsCRP, high-sensitivity C-reactive protein; SBP, systolic blood pressure; DBP, diastolic blood pressure.*

*q < 0.1.

### Microbial Clusters in Networks

**FIGURE 3:**
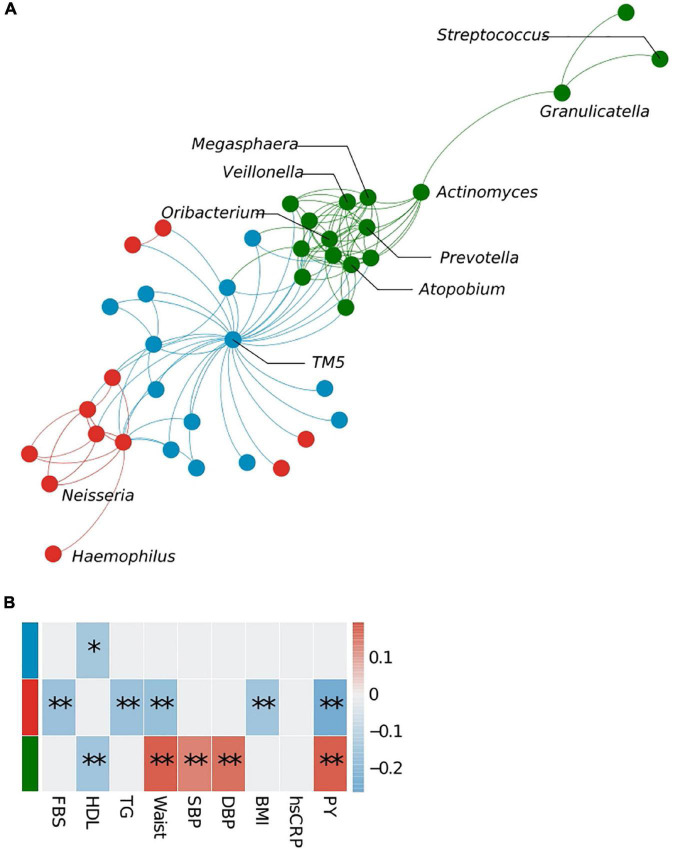
Bacterial cluster analysis in the sputum microbiota. **(A)** Microbial network at the genus level. Different colors represent different microbial clusters. **(B)** The Pearson correlation analysis of eigenvectors between each cluster and host clinical variables. Cluster colors (red, blue, and green) correspond with those shown in panels **(A,B)**. FBS, fasting blood sugar; HDL, high-density lipoprotein cholesterol; TG, triglycerides; SBP, systolic blood pressure; DBP, diastolic blood pressure; PY, pack-years. *p < 0.05, ^**^q < 0.1.

Microbe–microbe interactions were analyzed to determine which bacterial clusters are correlated with which host traits (Figure 3). Using the WGCNA approach, we identified three bacterial clusters and defined them using different colors (Supplementary Table 3). The green cluster, to which *Prevotella* belongs, correlated significantly with high-density lipoprotein cholesterol (HDL) levels (negative), waist circumference (positive), systolic blood pressure (positive), diastolic blood pressure (positive), and pack-years (PYs) (positive). The cluster containing *Neisseria* showed a negative correlation with fasting blood sugar (FBS), TG, waist circumference, body mass index (BMI), and PYs, whereas the blue cluster (represented by *TM5*) showed an association with HDL only. Bacterial network analysis also identified positive interactions between *Prevotella* and anaerobic bacteria such as *Megasphaera*, *Atopobium*, *Oribacterium*, *Actinomyces*, and *Veillonea* (green cluster).

### Metacommunities Within the Sputum Microbiota of an External Chronic Obstructive Pulmonary Disease Cohort

**FIGURE 4:**
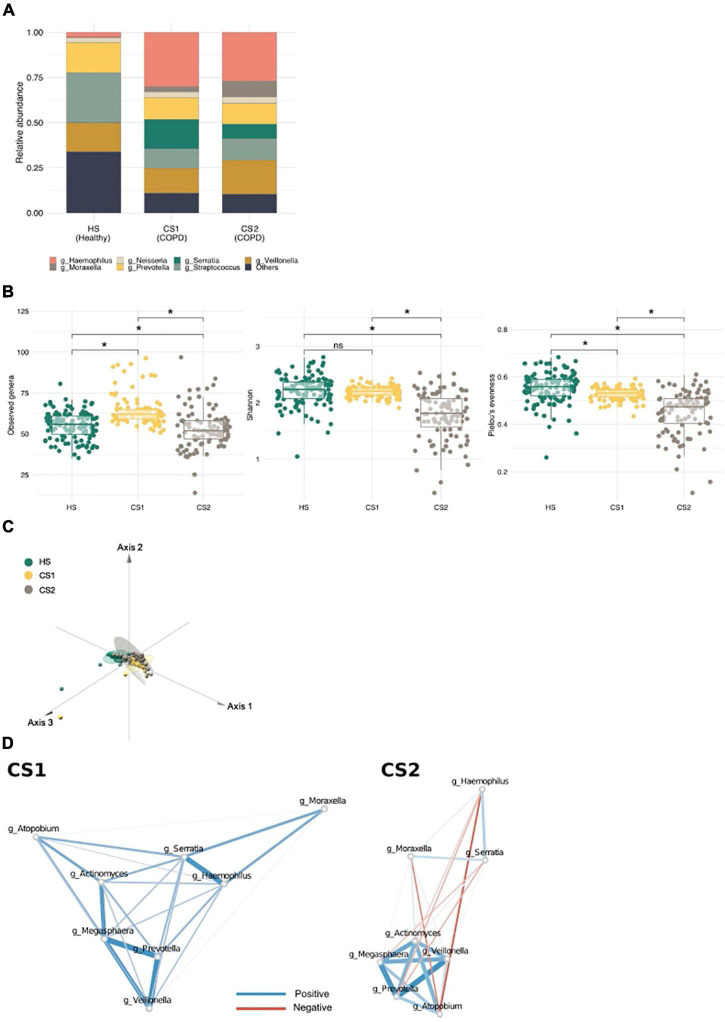
Metacommunities in the chronic obstructive pulmonary disease (COPD) cohort (Haldar et al.). **(A)** A stack bar graph showing the most abundant genera in each metacommunity. **(B)** Metacommunity diversity at the genus level. *q < 0.1. **(C)** A principal coordinate analysis (PCoA) plot showing the distribution of functional features between metacommunities. **(D)** The interaction of inflammatory bacteria with COPD-related taxa in CS metacommunities. Red and blue edges indicate negative and positive correlations, respectively. Edge width reflects the strength of the correlation.

To determine whether community typing is capable of distinguishing different COPD subtypes, we examined an external COPD cohort dataset (*n* = 324; healthy controls, *n* = 121; patients with COPD, *n* = 203) (Haldar et al., 2020). Again, we identified three main communities in this independent cohort; however, whereas all the healthy subjects (HSs) were grouped into one metacommunity (HS), and patients with COPD were subdivided into two (CS1 and CS2) (Figure 4A; Supplementary Figure 4A). The HS metacommunity was strongly driven by *Streptococcus*, as reported previously (Supplementary Figure 4B) (Haldar et al., 2020). CS1 and CS2 shared common driving bacteria: *Haemophilus*, *Veillonella*, *Streptococcus*, and *Prevotella* (Supplementary Table 4); however, CS1 was more strongly driven by *Serratia*, while CS2 was more strongly driven by *Moraxella*. Community diversity analysis showed that the richness of the CS1 metacommunity was significantly higher than that of the HS metacommunity, whereas the CS2 metacommunity showed significantly lower richness and evenness than the other two (the Kruskal–Wallis test, *q* < 0.1; Figure 4B). An analysis of the functional potential also revealed distinct functional clusters between the two COPD metacommunities (Pairwise Adonis test, *q* < 0.05; Figure 4C and Supplementary Table 5). Network analysis, performed to examine microbial interactions between metacommunities (Supplementary Figure 4D; Supplementary Tables 6–8), revealed that the number of interactions was higher in the COPD metacommunities (CS1 *n* = 1,107 and CS2 *n* = 1,188) than in the HS community (*n* = 269). Interestingly, the number of negative interactions also increased: 37.17, 49.86, and 52.19% in the HS, CS1, and CS2 metacommunities, respectively. Moreover, the *Prevotella* cluster (e.g., *Actinomyces*, *Atopobium*, *Veillonea*, and *Megasphaera*) identified in the Korean twin-family cohort showed changes in their interactions with COPD-related bacteria (e.g., *Haemophilus*, *Serratia*, and *Moraxella*) (Figure 4D). For example, in the CS1 metacommunity, the cluster maintained its positive correlation with the COPD taxa. However, in the CS2 metacommunity, *Prevotella*, *Megaspaera*, and *Atopobium* developed a negative correlation with *Serratia* and *Moraxella*. *Veillonella* maintained its positive correlation with *Moraxella* in both metacommunities. These differences in microbial interactions throughout COPD metacommunities point to the disease’s complexity, which may necessitate alternative therapeutic approaches depending on the subtype.

## Discussion

In this study, we used a community typing approach and identified three distinct metacommunities in the sputum microbiota of a healthy Korean twin-family cohort. These metacommunities were strongly driven by two dominant bacterial genera, *Prevotella* and *Neisseria*, and illustrated different ecological characteristics, as demonstrated by the community diversity. The N1 metacommunity consistently showed the greatest richness and evenness, whereas the *P* metacommunity occupied the other end of the spectrum. This lower community diversity was associated with host metabolic parameters similar to those observed in the gut microbiome in which reduced diversity indicates metabolic disorders. A previous study suggests that lower diversity in the sputum microbiome is associated with more severe bronchiectasis and defective lung function, as determined by FEV1 values (Dicker et al., 2021). Lower sputum bacterial diversity and evenness are also associated with an elevated level of proinflammatory cytokines (Durack et al., 2020). A link between reduced microbial diversity and impaired health could be induced by medications, host immune responses, or dominance by the pathogenic taxa (Faner et al., 2017). Therefore, the findings of reduced bacterial diversity in samples from subjects without any pulmonary disease suggest a microbial imbalance and potential associated immune changes.

Bacterial network analysis indicated potential adverse impacts driven by *Prevotella* and neighboring bacteria on the host health. *Megasphaera* (an anaerobe), *Atopobium* (a facultative anaerobe), *Oribacterium* (an anaerobe), *Actinomyce* (a facultative anaerobe), and *Veillonella* (an anaerobe), all of which were directly and strongly connected to *Prevotella*, are associated with smoking, lung cancer, and COPD (Morris et al., 2013; Lee et al., 2016; Wu et al., 2017). Given that the healthy lung provides an aerobic environment for the bacterial community (Huffnagle et al., 2017), anaerobic metabolism of bacteria coexisting with *Prevotella* supports their potential pathogenic effects in the lung. Moreover, the bacterial cluster that includes *Prevotella* showed more significant correlations with various host health parameters (i.e., HDL cholesterol level, waist circumference, blood pressure, and PY) than the *P* metacommunity. Therefore, we speculate that the negative associations of the *P* metacommunity with host physiology could be strongly influenced by specific bacterial interactions, with *Prevotella* as the keystone bacteria. Of note, we cannot exclude the possibility that such characteristics of the *P* metacommunity identified in this study may be induced by smoking, which has known adverse health effects.

We found it intriguing that respiratory *Prevotella* interacts with potential inflammatory bacteria, suggesting the potential to trigger local and systemic inflammatory conditions in a healthy population. A previous report suggests that *Prevotella* is associated with diseases at mucosal sites (i.e., periodontitis, complications related to rheumatoid arthritis, bacterial vaginosis, and metabolic disorders (Larsen, 2017). However, other studies show that *Prevotella* in the lung is protective because of its limited inflammatory capacity and its ability to increase pulmonary tolerance to respiratory pathogens (Bassis et al., 2015; Dickson et al., 2015). We found that all three metacommunities (i.e., N1, N2, and P) harbor *Prevotella* as one of the top driving bacteria, but with different abundance (about 20% abundance in N1 and N2 and 40% in P). *Prevotella* is a distinctive bacterium that lacks consensus regarding its role in inflammatory disease (Larsen, 2017). Given that our findings are based on a healthy population cohort, further studies are warranted to examine the association between host health dynamics and *Prevotella* abundance in the long term [including characterization at lower taxonomic levels (i.e., species or strain levels)].

Application of community typing to sputum samples collected from an external COPD cohort (Haldar et al., 2020) clearly distinguished HSs from patients; it also identified two sub-metacommunities within patients. Strong separation of the healthy population from diseased subjects confirms complete microbial dysbiosis in patients. Furthermore, the COPD sub-metacommunities themselves illustrated changes in the community diversity, functional potential, and pathogen (i.e., *Serratia* and *Moraxella*) abundance. Accordingly, we expected to find different degrees of disease severity between the two COPD metacommunities and assumed that the CS2 metacommunity would include patients with more severe COPD given its reduced microbial diversity and increased abundance of *Moraxella*. A previous study reported that *Moraxella catarrhalis* secretes an endonuclease distinct from that of *Serratia*, and *Moraxella* endonuclease was found to have enhanced cell–cell interactions and biofilm formation, which can aggravate disease conditions (Tan et al., 2019). Another previous study reported that patients with COPD with mild symptoms harbored only *Serratia*, not *Moraxella* (Kostadinova et al., 2015; Wang et al., 2016). Moreover, different numbers of coexclusions observed during network analysis of the two COPD metacommunities implies the competition triggered by environmental changes, such as disease severity. We observed that the microbial interactions, especially with the COPD pathogens, changed according to the COPD metacommunity. A recent study that identified subgroups of neutrophilic patients with COPD (i.e., neutrophilic balanced and neutrophilic *Haemophilus* subgroups) based on their sputum microbiome supports the possibility of distinguishing COPD severity using the microbiome data (Wang et al., 2021). The authors observed that the neutrophilic balanced subgroup had increased abundances of *Prevotella* and *Veillonella*, while these bacteria were lower in exacerbation subjects. Given that the microbial dynamics at deeper taxonomic levels change according to COPD severity (Wang et al., 2020), further studies should determine the taxa driving microbial interactions at the species or strain levels.

In conclusion, we showed that the cluster-based analysis (such as community typing) of the sputum microbiome can unmask key bacteria associated with host clinical phenotypes more effectively than the abundance-based approach; moreover, this approach shows a potential diagnostic and therapeutic application of the sputum microbiome to a heterogenic disease, such as COPD. COPD is a largely heterogeneous disease that shows a variable prognosis and response to drugs. Thus, microbial community typing of patients with COPD can help to identify those who share a similar microbial background and provide relevant targets to prevent disease exacerbation. Further studies are warranted to examine the long-term dynamics of *Prevotella* in host health conditions and the deeper links between distinct metacommunities and COPD phenotypes.

## Data Availability Statement

The datasets presented in this study can be found in online repositories. The names of the repository/repositories and accession number(s) can be found below: https://www.ebi.ac.uk/ena, ERP010734.

## Author Contributions

JS and YC analyzed the data. JS, JR, GK, and HY conceived the study objectives and study design. JS and HY prepared the draft manuscript. All authors contributed to the article and approved the submitted version.

## Conflict of Interest

GK is a founder of KoBioLabs Inc., a company that aims to characterize the role of host–microbiome interactions in chronic diseases. The remaining authors declare that the research was conducted in the absence of any commercial or financial relationships that could be construed as a potential conflict of interest.

## Publisher’s Note

All claims expressed in this article are solely those of the authors and do not necessarily represent those of their affiliated organizations, or those of the publisher, the editors and the reviewers. Any product that may be evaluated in this article, or claim that may be made by its manufacturer, is not guaranteed or endorsed by the publisher.
