# Supplementary material for: Sputum Bacterial Metacommunities in Distinguishing Heterogeneity in Respiratory Health and Disease
Source: Front Microbiol. 2022 Mar 31;13:719541. doi: 10.3389/fmicb.2022.719541 (PMC9008356; doi:10.3389/fmicb.2022.719541)
Supplement: Supplementary file 2 [file Data_Sheet_1.pdf]

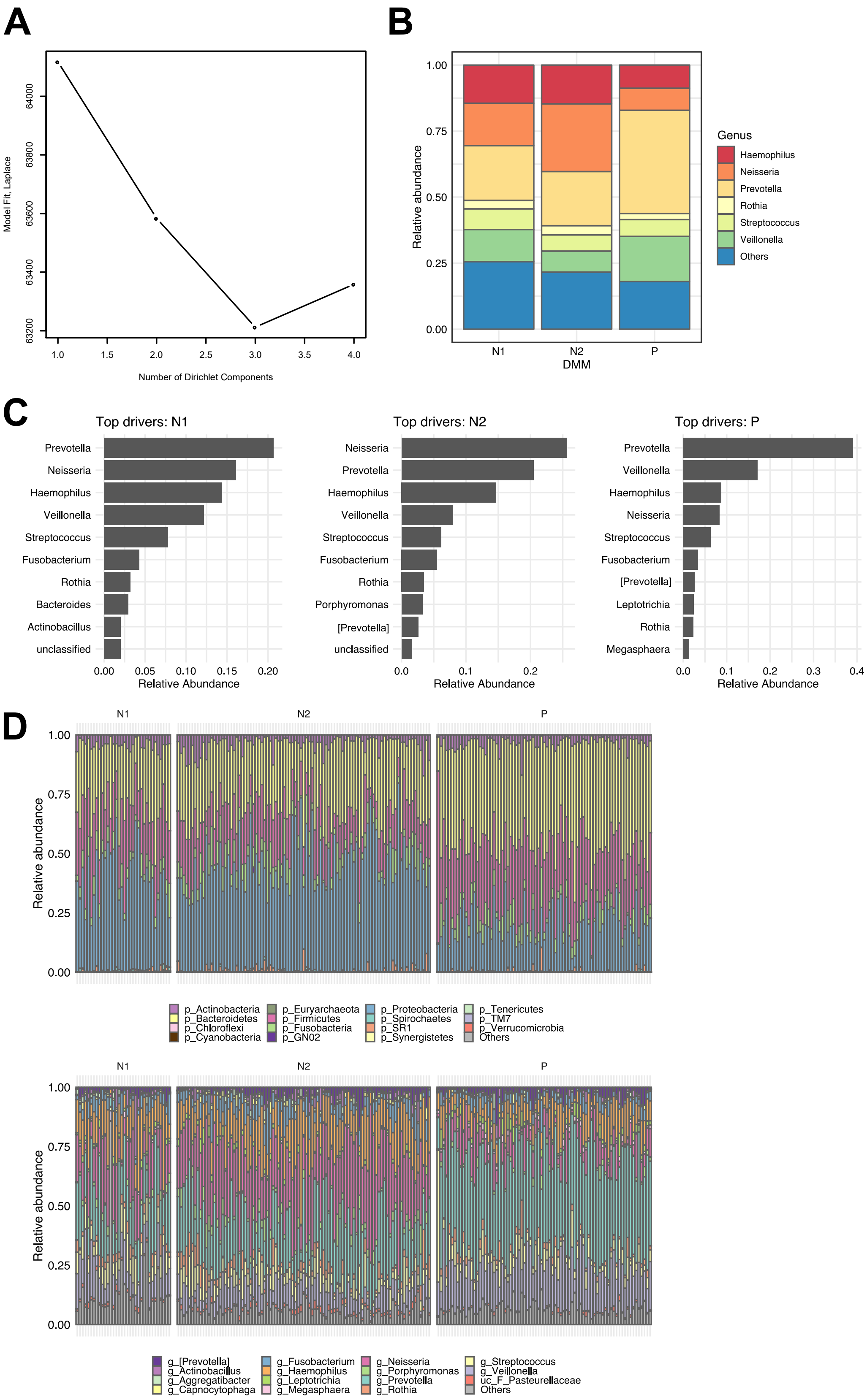
A
Model Fit, Laplace
63200
63400
63600
63800
64000
1.0
1.5
2.0
2.5
3.0
3.5
4.0
Number of Dirichlet Components
B
Relative abundance
0.00
0.25
0.50
0.75
1.00
N1
N2
P
DMM
Genus
Haemophilus
Neisseria
Prevotella
Rothia
Streptococcus
Veillonella
Others
C
Top drivers: N1
Prevotella
Neisseria
Haemophilus
Veillonella
Streptococcus
Fusobacterium
Rothia
Bacteroides
Actinobacillus
unclassified
0.00
0.05
0.10
0.15
0.20
Relative Abundance
Top drivers: N2
Neisseria
Prevotella
Haemophilus
Veillonella
Streptococcus
Fusobacterium
Rothia
Porphyromonas
[Prevotella]
unclassified
0.0
0.1
0.2
Relative Abundance
Top drivers: P
Prevotella
Veillonella
Haemophilus
Neisseria
Streptococcus
Fusobacterium
[Prevotella]
Leptotrichia
Rothia
Megasphaera
0.0
0.1
0.2
0.3
0.4
Relative Abundance
D
N1
N2
P
Relative abundance
0.00
0.25
0.50
0.75
1.00
p_Actinobacteria
p_Bacteroidetes
p_Chloroflexi
p_Cyanobacteria
p_Euryarchaeota
p_Firmicutes
p_Fusobacteria
p_GN02
p_Proteobacteria
p_Spirochaetes
p_SR1
p_Synergistetes
p_Tenericutes
p_TM7
p_Verrucomicrobia
Others
N1
N2
P
Relative abundance
0.00
0.25
0.50
0.75
1.00
g_[Prevotella]
g_Actinobacillus
g_Aggregatibacter
g_Capnocytophaga
g_Fusobacterium
g_Haemophilus
g_Leptotrichia
g_Megasphaera
g_Neisseria
g_Porphyromonas
g_Prevotella
g_Rothia
g_Streptococcus
g_Veillonella
uc_F_Pasteurellaceae
Others

Supplementary Figure 1. Community typing of sputum samples from a healthy population. (A) Evaluation of model fit using Laplace approximation. **(B)** Top six abundant genera in metacommunities. **(C)** The driving genera in each metacommunity. N1: Neisseria 1; N2: Neisseria 2; P: Prevotella metacommunity. **(D)** Microbial composition at the phylum (top) and genus (bottom) level.

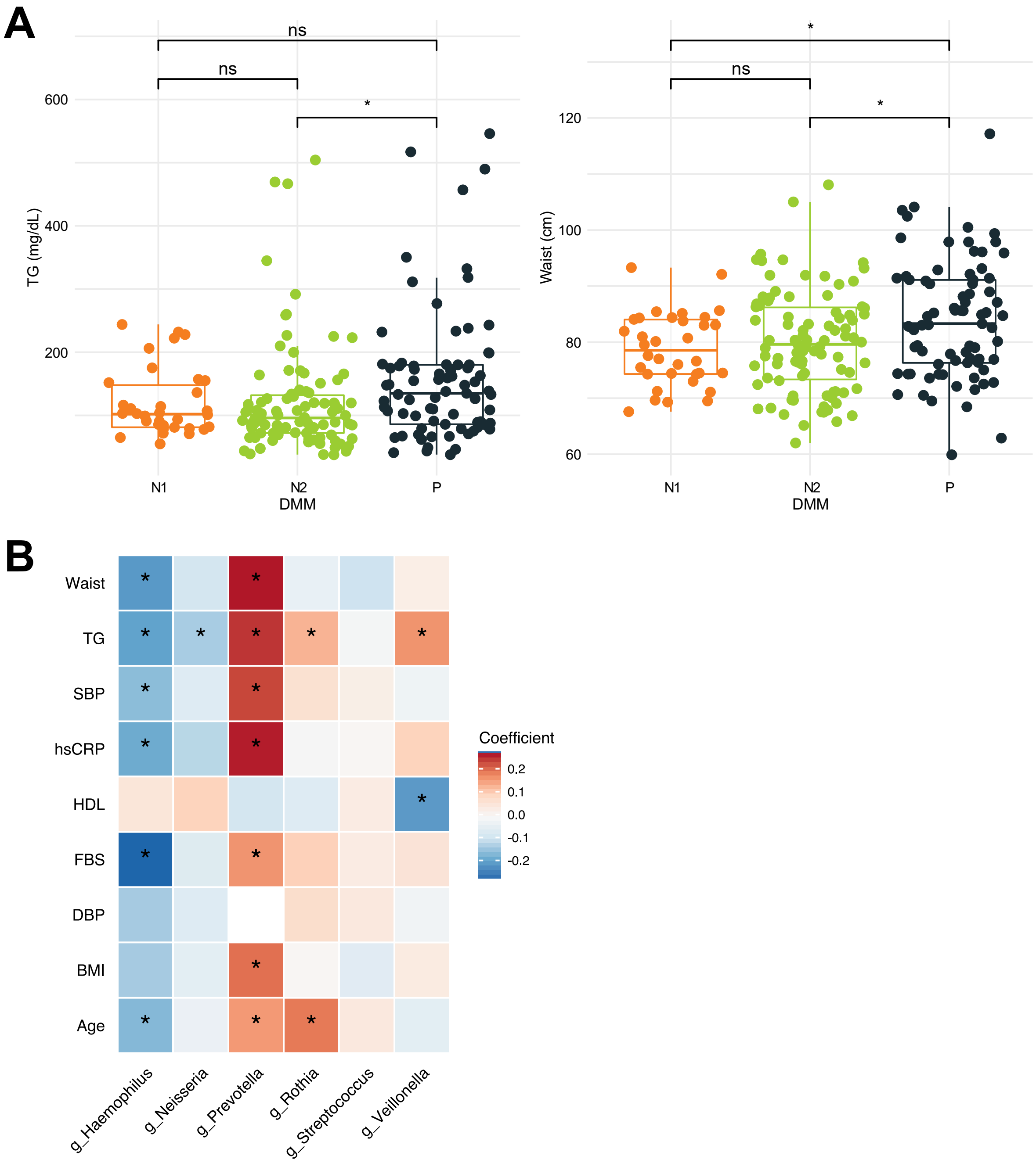

Supplementary Figure 2. Associations with clinical variables. (A) Metacommunities. TG: triglycerides. **(B)** Top six abundant genera in metacommunities. * q-value < 0.1.

A

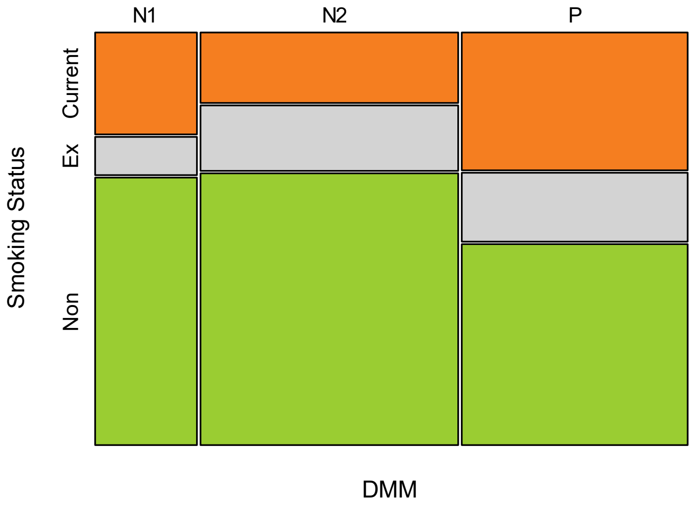

	Smoking Status				
	**Current**	**Ex**	**Non**	**Total**	**p-value**
N1	8 (17.4%)	3 (10.7%)	21 (19.1%)	32	> 0.05
N2	14 (30.4%)	13 (46.4%)	54 (49.1%)	81	
P	24 (52.2%)	12 (42.9%)	35 (31.8%)	71	
Total	46	28	110	184	

B

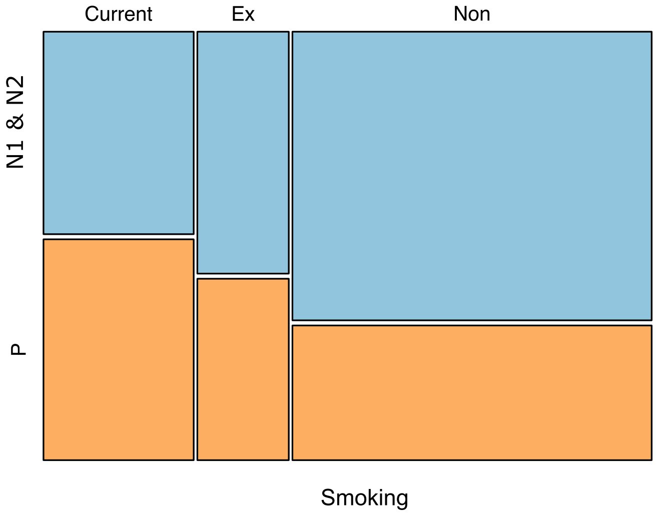

	Smoking Status				
	**Current**	**Ex**	**Non**	**Total**	**p-value**
N1 &N2	22 (47.8%)	16 (57.1%)	75 (68.2%)	113	0.052
P	24 (52.2%)	12 (42.9%)	35 (31.8%)	71	
Total	46	28	110	184	

Supplementary Figure 3. Association between host smoking and metacommunities. (A) Metacommunity distribution and host smoking status. Orange: current-smoker; gray: ex-smoker; green: non-smoker. **(B)** Sub-division of the metacommunity and connections with host smoking status.

frontiers

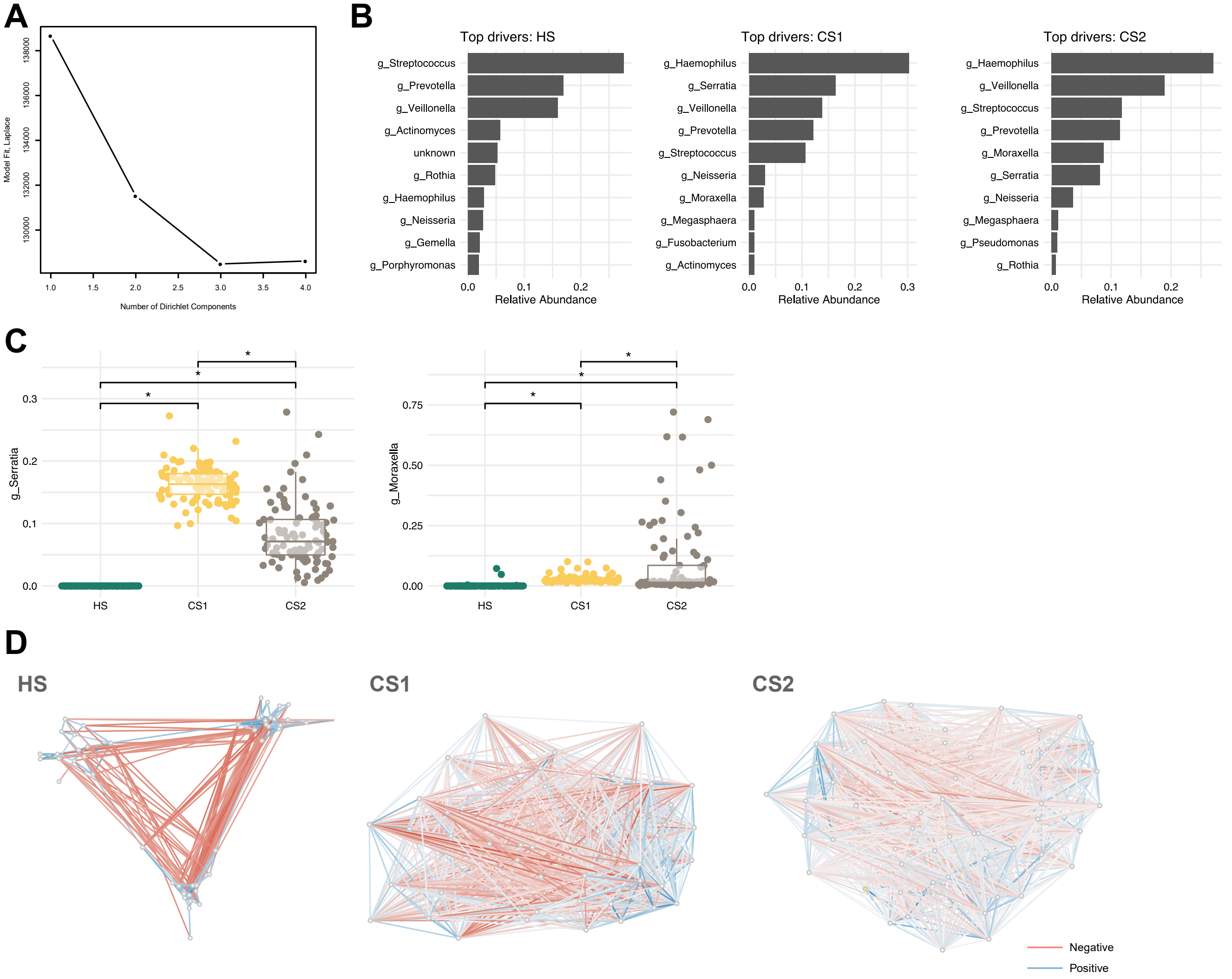
A
Model Fit, Laplace
Number of Dirichlet Components
B
Top drivers: HS
g_Streptococcus
g_Prevotella
g_Veillonella
g_Actinomyces
unknown
g_Rothia
g_Haemophilus
g_Neisseria
g_Gemella
g_Porphyromonas
Relative Abundance
Top drivers: CS1
g_Haemophilus
g_Serratia
g_Veillonella
g_Prevotella
g_Streptococcus
g_Neisseria
g_Moraxella
g_Megasphaera
g_Fusobacterium
g_Actinomyces
Relative Abundance
Top drivers: CS2
g_Haemophilus
g_Veillonella
g_Streptococcus
g_Prevotella
g_Moraxella
g_Serratia
g_Neisseria
g_Megasphaera
g_Pseudomonas
g_Rothia
Relative Abundance
C
g_Serratia
g_Moraxella
HS
CS1
CS2
D
HS
CS1
CS2
Negative
Positive

Supplementary Figure 4. Community typing of sputum samples from an independent COPD dataset (Haldar et al., 2020). (A) Evaluation of model fit using Laplace approximation (for both healthy and COPD individuals). **(B)** Driving genera in the metacommunity. **(C)** Abundance of Serratia and Moraxella in metacommunities. **(D)** Changes in microbial interactions.
